# Sepsis and obesity: a scoping review of diet-induced obesity murine models

**DOI:** 10.1186/s40635-024-00603-0

**Published:** 2024-02-23

**Authors:** Mikaela Eng, Keshikaa Suthaaharan, Logan Newton, Fatima Sheikh, Alison Fox-Robichaud, the National Preclinical Sepsis Platform, Sepsis Canada

**Affiliations:** 1 Thrombosis and Atherosclerosis Research Institute (TaARI), Hamilton, Canada; 2 Department of Medicine, Faculty of Health Sciences, McMaster University, Hamilton, Canada; 3 Department of Health Research Methods, Evidence and Impact, Faculty of Health Sciences, McMaster University, Hamilton, Canada; 4 Division of Critical Care, Department of Medicine, Faculty of Health Sciences, McMaster University, Hamilton, Canada

**Keywords:** Animal model, Infection, Mouse, Diet-induced obesity, Organ function

## Abstract

**Background:**

Sepsis, the life-threatening host response to infection, is a major cause of mortality. Obesity increases vulnerability to sepsis; however, some degree of obesity may be protective, called the “obesity paradox”. This scoping review systematically maps the literature on outcomes associated with diet-induced obesity and sepsis-induced organ injury, focusing on non-transgenic murine models.

**Methods:**

A literature search of primary articles was conducted from database inception to June 2023. Eligible articles compared diet-induced obesity to non-obese mice in sepsis models involving live pathogens. Two reviewers screened articles and extracted data on obesogenic and sepsis models utilized, and organ injury outcomes, including physiological dysfunction, histological alterations, and biochemical changes.

**Results:**

Seventeen studies met eligibility criteria; 82% used male C57BL/6 mice, and 88% used cecal ligation and puncture to induce sepsis. Most studies used 60% high-fat diets compared to 10–16% fat in controls. Seven (64%) studies reported increased mortality in obese septic mice, one (9%) observed a decrease, and three (37%) found no significant difference. The liver, lungs, and kidneys were the most studied organs. Alanine transaminase results were inconclusive. Myeloperoxidase levels were increased in the livers of two studies and inconclusive in the lungs of obese septic mice. Creatinine and neutrophil gelatinase-associated lipocalin were elevated in obese septic mice.

**Conclusions:**

There is variability in the methodology and measured outcomes in murine models of diet-induced obesity and sepsis and a lack of studies in female mice. The absence of standardized models has produced conflicting findings on the impact of obesity on sepsis outcomes.

**Supplementary Information:**

The online version contains supplementary material available at 10.1186/s40635-024-00603-0.

## Take-home message

This scoping review highlights the varied use of murine models in studying sepsis and obesity's effects on organ injury, leading to inconsistent data and hindering progress. Standardizing mouse models, incorporating both sexes, and agreeing on outcome measures are essential for enhancing comprehension of obesity's influence on sepsis response.

## Introduction

Sepsis, the life-threatening response to infection resulting in organ damage and dysfunction, is the leading cause of death worldwide [1, 2]. The substantial healthcare burden is evident, with intensive care unit (ICU) stays costing billions in Canada [3]. Despite advancements in sepsis understanding, treatment remains supportive due to the diverse disease trajectory among patients. Co-existing conditions such as obesity, diabetes, heart disease, renal failure, and alcohol use disorder further complicate sepsis, altering the immune response [4]. Yet, the specific impact of these co-morbidities on sepsis outcomes remains elusive.

Obesity, characterized by a BMI exceeding 30, is a widespread issue globally, disregarding socioeconomic differences [5]. Overweight and obese patients are increasingly represented in critical care, accounting for a significant proportion of ICU admissions [6]. Paradoxically, observational studies suggest that obesity might confer a survival advantage in sepsis, defying conventional health implications [7–10]. However, pre-clinical research outcomes on obesity's influence in sepsis are inconsistent, hampering effective translation to clinical practice. It imperative to establish relevant models mimicking human scenarios to unravel obesity's intricate role, encompassing its impact on sepsis occurrence, organ dysfunction, and mortality. Such insights hold the key to innovative therapeutic strategies in sepsis management.

Murine models are pivotal for comprehending sepsis–obesity dynamics, driven by their simplicity, reproducibility, and cost-effectiveness in sepsis research [11, 12]. The established "gold-standard" sepsis model is cecal ligation and puncture (CLP), involving cecal puncture and fecal introduction into the peritoneal cavity [13]. Another model, fecal-induced peritonitis (FIP), injects bacterial inoculum from a donor animal's cecal contents into the peritoneal cavity [14]. Murine obesity research employs genetic (monogenic or polygenic) or non-genetic models, such as diet-induced obesity (DIO). While genetic models unravel gene mechanisms, they might lack translational relevance due to rare or non-existent human-equivalent mutations [15]. In contrast, DIO mirrors human dietary imbalances contributing to obesity more faithfully. However, the lack of consensus on optimal pre-clinical model combinations leads to conflicting findings and literature gaps.

This scoping review aims to comprehensively explore the literature on the effects of live pathogens in murine models of diet-induced obesity (DIO) and sepsis, with the objective of systematically assessing and synthesizing available research to elucidate the impact of DIO on sepsis-related organ injury. Additionally, this review intends to evaluate methodological aspects and identify knowledge gaps, thereby contributing to the enhancement of research quality and understanding.

## Methods

This scoping review adheres to the PRISMA–ScR guidelines [16] and follows a five-stage process based on the framework by Arksey and O’Malley [17], as well as advancements by Levac et al. [18]. The stages encompassed defining the research question, identifying pertinent studies, selecting studies, data charting, and summarizing and reporting results. The review's protocol is available on Open Science Framework with the identifier 10.17605/OSF.IO/FE7KY.

### Stage 1: identifying a research question

- Primary: In murine models of DIO and pathogen-driven sepsis, what are the reported outcomes on the impact of obesity on sepsis-induced organ injury?
- Secondary: In murine sepsis models, is there evidence that DIO protects against sepsis-induced organ dysfunction?

### Stage 2: identifying relevant studies

Relevant studies were identified by searching PubMed, Medline, EMBASE, Web of Science, and CINAHL from inception to June 2023. Search terms included sepsis, septicemia, bacteremia, murine model, mouse model, obesity, and high-fat diet. The search terms were adapted to each database as needed. Additional file 1 presents a sample search strategy.

### Stage 3: study selection

Relevant studies were screened by title and abstract, followed by full-text review using Covidence (Veritas Health Innovation, Melbourne, Australia) [19]. Two reviewers conducted independent screenings, resolving discrepancies through discussion or a third reviewer's input.

A modified SYRCLE tool with 21 sub-items was used, (excluding sub-item 17 due to lack of relevance) (Additional file 2: Table S1). This aimed to evaluate each study's quality, bias, strengths, and limitations in murine sepsis and obesity research. Despite its uncommon use in scoping reviews, risk of bias assessment was conducted to enhance discussions on study quality and inform future research, involving two independent reviewers and resolving disagreements through a third reviewer's consultation.

### Stage 4: eligibility

This scoping review included non-transgenic murine models investigating the impact of high-fat and/or diet-induced obesity (DIO) on sepsis outcomes. Eligible sepsis models encompassed bacterial sepsis, polymicrobial sepsis, and cecal ligation and puncture. Included studies explored histological, biochemical, physiological, and immune changes associated with organ injury. Excluded were studies involving humans, rats, other animal models, lipopolysaccharide sepsis models, obesity knock-out models (*ob/ob, db/db*), and solely in vitro approaches. Publications such as editorials, abstracts, commentaries, letters, systematic reviews, and meta-analyses were excluded, though their reference lists were reviewed for relevant articles.

### Stage 5: charting the data

Key information from the included studies was abstracted, independently and in duplicate, using standardized data abstraction forms (Additional file 1: Data extraction file). The following information was extracted:

1. Author(s).
2. Year of publication.
3. Country of publication.
4. Breed, supplier, sex, and age of mice.
5. Organs evaluated.
6. Type of diet (composition, percent of kcal).
7. Method of diet delivery.
8. Length of time on the diet.
9. Body weight and fat mass.
10. The method by which sepsis was induced, site of infection, and dose.
11. Endpoint time.
12. Antibiotics, fluids, and analgesia.
13. Outcomes including glucose and insulin response, mortality, biomarkers of organ dysfunction, myeloperoxidase, and cytokine changes.

The data abstraction form was tested on three studies, and then data extraction was conducted independently and in duplicate by two reviewers, with discrepancies resolved through discussion or third-party arbitration.

### Stage 6: collating, summarizing, and reporting the results

The study presented results summarizing the impact of DIO on sepsis outcomes, using tables to organize bibliographic, obesogenic, and sepsis model data. The narrative synthesis highlighted DIO model development, sepsis induction methods, and outcomes, assessing whether DIO offers sepsis protection. Similar outcome studies reported in parallel, and conflicting evidence was compared.

## Results

**Fig. 1:**
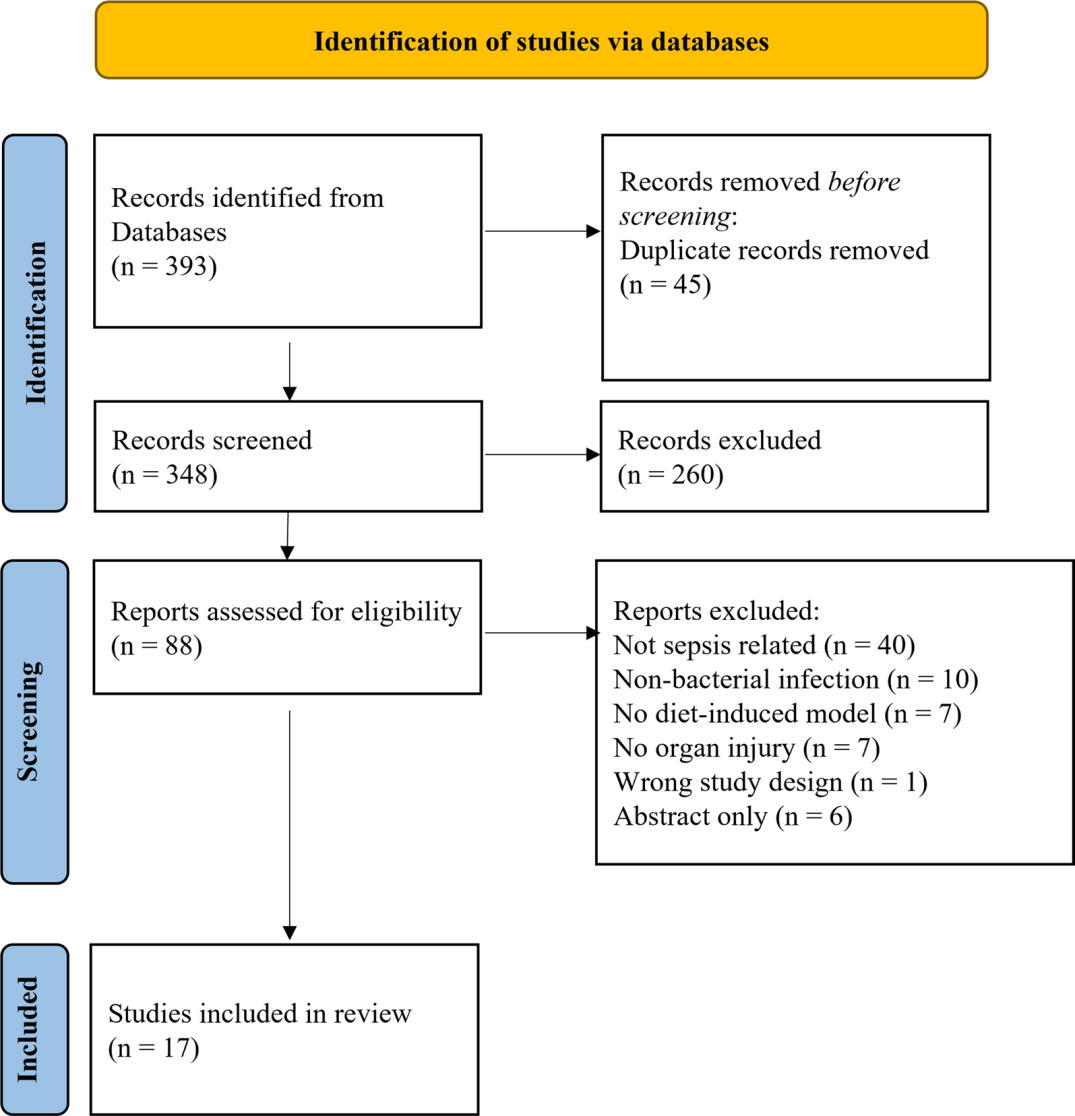
Preferred Reporting Items for Systematic Reviews and Meta-Analyses (PRISMA) Flow Chart. A chart representation of the process used to collect relevant literature from a set of databases and criteria. Beginning with the identification process, this chart displays how 393 collected studies were screened to determine which articles should be included in the review. The screening and inclusion process provided a total of 17 studies that met all the desired criteria

A total of 393 articles were initially identified through the search. After removing duplicates, 348 articles underwent initial screening, resulting in 88 articles based on title and abstract. Following further evaluation, 71 articles were excluded for not meeting inclusion criteria, leading to a final selection of 17 articles that met the criteria (Fig. 1) [20–36].

### Study characteristics

Table 1 summarizes the characteristics of the 17 included studies, originating from eight different countries, predominantly the United States (41%). Most studies (88%) utilized male C57BL/6 background mice, while exceptions included one study (6%) involving male Swiss mice [31], another (6%) with 57BL/6JRj mice [23], and one study (6%) exclusively using female mice [22]. Mice ages ranged from three to 24 weeks, with a notable proportion (53%) initiating diet at 6 weeks [20, 21, 24, 25, 27, 29, 32, 34, 35]. Most studies obtained mice from commercial suppliers, although three (18%) employed in-house bred mice [22, 28, 31], and another three (18%) did not specify the source [29, 33, 36]. The liver was the most frequently evaluated organ (71%), followed by the lungs (29%) and kidney (24%).

**Table 1:** Summary of study characteristics

Author	Country	Mouse strain	Supplier	Sex	Age at start of diet (weeks)	Organs evaluated
DeMartini et al. [20]	USA	C57BL/6	Charles River	M	6	Heart
Frydrych et al. [21]	USA	C57BL/6	Jackson	M	6	Bone marrow, spleen
Gomes et al. [22]	Brazil	C57BL/6	In-house	F	3–4	Liver
Goossens et al. [23]	Belgium	57Bl/6JRj	Janvier	M	24	Muscle
Kaplan et al. [24]	USA	C57BL/6	Charles River	M	6	Liver, lung, spleen
Kaplan et al. [25]	USA	C57BL/6	Charles River	M	6	Liver
Khan et al. [26]	Canada	C57BL/6	Taconic	M	3–5	Liver, lung
Lewis et al. [27]	USA	C57BL/6	Jackson	M	6	Kidney, liver
Panpetch, et al. [28]	Thailand	C57BL/6	In-house	M	8	Intestines, kidney, liver
Rivera et al. [29]	USA	C57BL/6	NR	M	4–6	Liver
Siegl et al. [30]	Germany	C57BL/6	Janvier	M	7	Liver, lung
Souza et al. [31]	Brazil	Swiss	In-house	M	8	Hypothalamus, liver, spleen
Strandberg et al. [32]	Sweden	C57BL/6	Harlan	M	6–8	Kidney, liver, spleen
Su et al. [33]	Taiwan	C57BL/6	NR	M	5	Kidney
Vankrunkelsven et al.[34]	Belgium	C57BL/6	Janvier	M	6	Liver, muscle
Williamson et al.[35]	USA	C57BL/6	Charles River	M	6	Liver, lung
Yeh et al. [36]	Taiwan	C57BL/6	NR	M	5	Lung

*NR* not reported, *USA* United States of America, *HFD* high-fat diet, *LFD* low-fat diet

### Models of sepsis

Table 2 provides an overview of the sepsis induction methods utilized, with 82% of studies employing cecal ligation and puncture (CLP) [20–26, 28–31, 33–36]. The prevalent CLP techniques included double-puncture with a 22G needle (18%) [20, 25, 35] and single puncture using a 23G needle (18%) [30, 33, 36]. A live-bacteria model was used in one (6%) study [32], and another (6%) [27] induced sepsis with fecal slurry. Post-sepsis evaluations were conducted between 6 h and 28 days, with 76% of studies not reporting antibiotic use [21, 22, 24–33, 36]. However, three (18%) studies [20, 34, 35] administered imipenem, one (6%) in combination with cilastatin [34]. One study (6%) [23] mentioned antibiotic use without specifying type or dosage. Fluid resuscitation was performed in 71% of studies [20, 22–26, 29, 30, 33–36], commonly using either 0.6 ml or 1 ml of saline (35%) [20, 24, 25, 29, 30, 35]. Conversely, analgesics were not used throughout the sepsis timeline in 65% of studies [20–22, 24, 25, 27, 29–32, 35].

**Table 2:** Summary of characteristics of sepsis model

Authors	Method of sepsis	Route of infection	Dose	Endpoint (h)	Antibiotics	Fluids	Analgesia
DeMartini [24]	CLP	IP	Double puncture; 22G	6	Imipenem (25 mg/kg)	Sterile saline, SC; (0.6 mL)	NR
Frydrych [25]	CLP	IP	Double puncture; 20G	28 days	NR	NR	NR
Gomes [26]	CLP	IP	Single puncture; 21G	7 days	NR	Sterile saline, SC; (0.5 mL/10 g)	NR
Goosses [27]	CLP	IP	Needle not specified	1, 5 days	Yes^a^	Yes^a^, IV	Yes^a^
Kaplan [28]	CLP	IP	Double puncture; 21G	1–30	NR	Sterile saline, SC; (0.6 mL)	NR
Kaplan [29]	CLP	IP	Double puncture; 22G	6	NR	Sterile saline, SC; (0.6 mL)	NR
Khan [30]	CLP	IP	Single puncture; 18G	6	NR	LR, SC before surgery; (2 mL) LR, IV^b^ and 4 h later	Yes^a^
Lewis [31]	CS	IP	500 μL of CS in 10% glycerol	14 days	NR	NR	NR
Panpetch [32]	CLP	IP	Double puncture; 21G	24	NR	NR	Fentanyl, SC^b^ and 6 h; (0.03 mg/kg)
Rivera [33]	CLP	IP	Triple puncture; 20G	6	NR	Saline^b^; (1 mL)	NR
Siegl [34]	CLP	IP	Single puncture; 23G	10 days	NR	Sterile saline, SC^b^; (1 mL)	NR
Souza [35]	CLP	IP	NR	24	NR	NR	NR
Strandberg [36]	S. *aureus*	IV	5 × 10^7^ CFU	24, 5–7 or 17 days	NR	NR	NR
Su [37]	CLP	IP	Single puncture; 23G	12, 24	NR	Sterile saline, SC^b^; (4 mL/kg)	0.25% bupivacaine; (100 µL)
Vankrunkelsven [38]	CLP	IP	18G	5 days	Imipenem/Cilastatin	IV^a^	Buprenorphine
Williamson [39]	CLP	IP	Double puncture; 22G	18	Imipenem (25 mg/kg)	Normal saline, SC; (1 mL)	NR
Yeh [40]	CLP	IP	Single puncture; 23G	12, 24	NR	Sterile saline, SC^b^; (4 mL/kg BW)	0.25% bupivacaine; (100 µL)

*NR* not reported, *MRSA* methicillin-resistant *Staphylococcus aureus, IV* intravenous, *SC* subcutaneous, *CLP* cecal ligation and puncture, *IP* intraperitoneal, *CS* cecal slurry, *LR *lactated ringers, *BW* body weight

^a^No further specifications given

^b^Given post-surgery

### Models of obesity

Table 3 summarizes the obesity models employed in the studies, with 53% using a high-fat diet comprising 60% kilocalories (kcal) of fat [20, 21, 24, 25, 27, 31, 33, 35, 36]. Other studies specified diet composition in terms of percent butterfat [26], gram percent fat [32], g/kg of butterfat [29], kilojoule % of fat [34], w/w [28], or percent lipids not converted to %kcal fat [22]. Control diets ranged from 10 to 16% kcal of fat, and 24% of studies used normal or standard chow [26, 31, 33, 36] as controls. Diet duration varied from 3 days to 27 weeks, with 6 [25, 26, 35] or 12 weeks [23, 30, 34] being common. Mice on high-fat diets typically exhibited increased body weight or fat mass at the study’s end [20, 22, 24–30, 32–36], although 65% did not report fat mass [21–24, 26, 28–31, 35, 36], and 18% did not report body weight or fat mass [21, 23, 31]. While high-fat diets were used across all studies, two (12%) also employed genetic knock-out models for obesity induction [32, 34], with this review focusing exclusively on high-fat diet-induced obesity models.

**Table 3:** Summary of characteristics of the obesity model

Authors	HFD (%kcal fat)	LFD (%kcal fat)	Time on diet (weeks)	Method of feeding	Body weight^a^ (g; SD)	Fat mass^a^ (g; SD)	Model of obesity
DeMartini [24]	60	16	5	Ad libitum	HFD:36.3(34.2–38.1 IQR) LFD:27.8(27.0–28.4 IQR)	HFD: 8.0 ± 2.6 LFD: 0.6 ± 0.5	HFD
Frydrych [25]	60	13	22–26	Ad libitum	NR	NR	HFD
Gomes [26]	19.55% lipids	4.45% lipids	14	Ad libitum	HFD 25.69 ± 3.12 LFD 21.93 ± 1.57	NR	HFD
Goossens [27]	45	10	12	Ad libitum	NR	NR	HFD
Kaplan [28]	60	16	3	Ad libitum	HFD: 25.2 g ± 0.4 LFD:23.4 g ± 0.4	NR	HFD
Kaplan. [29]	60	16	6–7	Ad libitum	Increased^b^	Increased^b^	HFD
Khan [30]	21% butterfat	NC	6, 15, 27	Ad libitum	HFD: 50.8 g ± 1.05 LFD^a^: 39.6 ± 1.18	NR	WD
Lewis [31]	60	10	20–21	Ad libitum	HFD: 46.6 ± 4.53 LFD: 32.3 ± 2.08	HFD: 16.0 ± 5.21 LFD: 4.1 ± 1.30	HFD
Panpetch [32]	60%w/w	4.5% w/w	20	Ad libitum	Increased^b^	NR	HFD
Rivera [33]	50 g/kg butterfat	15 g/kg butterfat	3	Ad libitum	Twofold higher^b^	NR	WD
Siegl [34]	50	11	12	Ad libitum	HFD: 34.4 ± 0.5 LFD: 27.7 ± 0.2	NR	HFD
Souza [35]	60	SC	3 days	Ad libitum	NR	NR	HFD
Strandberg [36]	34.9/35.9 g% fat	4.0/4.3 g% fat	8	Ad libitum	HFD: 39.3 ± 1.1 LFD 28.8 ± 0.5	HFD: 17.0 ± 0.6 LFD: 5.5 ± 0.2	HFD, *ob/ob*
Su [37]	60	SC	10	Ad libitum	HFD: 36.5 ± 1.1 LFD: 26.4 ± 0.7	HFD: 2.54 ± 0.09 LFD: 0.63 ± 0.02	HFD
Vankrunkelsven [38]	60 kJ%	9 kJ%	11–12	Ad libitum	HFD: 43.9 ± 4.7 LFD: 30.2 ± 1.9	Increased^b^	HFD, *ob/ob*
Williamson [39]	60	16	6–7	Ad libitum	Increased^b^	NR	HFD
Yeh [40]	60	SC	10	Ad libitum	HFD: 41.5 ± 1.3 LFD: 27.7 ± 1.5	NR	HFD

*NR* not reported, *NC* normal chow, *SC* standard chow, *ob/ob* leptin-deficient mice, *HFD* high-fat diet, *LFD* low-fat diet, WD western diet

^a^Reported as mean unless otherwise stated

^b^HFD vs. LFD

^c^30% diet restriction at 12 weeks in LFD group only

^d^Calorie restriction at 10 weeks

### Key findings

The key outcomes of each study are summarized in Table 4. Six (35%) studies reported glucose intolerance before sepsis induction [22, 25–28, 34]. Of those studies, five (83%) found that obese mice had significantly higher glucose levels than their non-obese counterparts, while one (17%) reported no difference [22]. There were inconsistent results when reporting the impact of a high-fat diet on sepsis mortality. Eleven out of the 17 (65%) studies [21–25, 27, 28, 30–32, 34] reported mortality. Of these studies, seven (64%) reported an increase in mortality in their obese septic mice [21, 22, 24, 25, 30–32], one (9%) observed a decrease in mortality [27], and three (27%) studies did not see any difference [23, 28, 34]. Among the investigations that documented elevated mortality in obese septic, four studies (57%) utilized saline for fluid resuscitation; however, none of them reported the administration of antibiotics or analgesics [22, 24, 25, 30]. The study that observed a reduction in mortality did not document the use of fluid resuscitation, antibiotics nor analgesics [27]. Of the three studies that reported no discernible difference in mortality, two studies (66%) disclosed the utilization of fluid resuscitation without specifying the type or volume of fluids [23, 34], while the final publication failed to mention any use of fluid resuscitation [28]. Intriguingly, all three papers that did not identify a disparity in mortality were the only studies to reporting mortality outcomes and analgesic usage [23, 28, 34]. Finally, the use of antibiotics and mortality were only reported in two studies [23, 34] in which both studies observed no difference in mortality. Vankrunkelsven [34] reported the use of imipenem/cilastin and Goossens [23] reported the use of antibiotics without disclosing further information.

**Table 4:** Outcomes of the impact of obesity on sepsis

Author	Blood glucose (mmol/L; IQR)^i^	Mortality (n = %)^a^	Biomarkers of organ dysfunction (SD)	MPO (U/100 mg tissue; SD)	IL-6	TNFα
DeMartini [24]	NR	NR	ND cTNI 6 h (plasma)	↑ (heart)^a,b^	NR	NR
Frydrych [25]	NR	↑ LFD-S: 20% HFD-S: 60%	NR	NR	↑ 6 h (blood)^a^	↓ 18 h (blood)^a^
Gomes [26]	ND^h^ LFD: 20.18 (15.35–23.15) HFD:29.08 (22.77–32.74) LFD-S: 17.03 (14.95–18.03) HFD-S:21.24 (14.24–25.64)	↑ LFD-S: 23.8% HFD-S: 41.7%	ND ALT (serum; U/L): LFD: 22.01 (22.01–88.05 IQR) HFD: 200.3 (123.3–277.4 IQR) LFD-S: 202.5 (168.4–231.1 IQR) HFD-S: 224.5 (154.1–306 IQR) ↑ Liver histology score^b^	NR	↑ (serum)^a^ ↑ (liver)^b^	↑ (serum)^a,b^ ↑ (liver)^b^
Goossens [27]	NR	ND LFD-S: 17% HFD-S: 17%	↑ atrophy(muscle)^b^	NR	NR	NR
Kaplan [28]	NR	↑	↑ lung injury score^a^ HFD: 3.5 ± 0.5 AU HFD-S: 8.6 ± 0.9 AU	↑ 3h^b^, 6 ha^b^, 18 h^a,^^b^ (liver) ↑ 1 h^a,^^b^, 3 h^b^, 6 h^a,^^b^, 18 h^a^ (lung)	↓ 3 h^a,^^b^ (plasma) ↑ 1 h^b^, 3 h^b^, 6 h^b^, 18 h^b^ (plasma)	↓ 3 h^a^ (plasma) ↑ 6 h^b^ 18 h^b^ (plasma)
Kaplan [29]	↑^e^	↑	↑ ALT (U/L; plasma)^a,^^b^ LFD: 88 ± 21 HFD: 63 ± 4 LFD-S: 154 ± 10 HFD-S: 227 ± 32	↑6 h (liver)^a,b^ LFD-S: 7 ± 0.3 HFD-S: 11.4 ± 1.4	↑ (plasma; U/L)^a,b^ LFD: 88 ± 21 HFD: 63 ± 4 LFD-S: 154 ± 10 HFD-S: 227 ± 32	↓ (plasma)^a^ ↑ (plasma)^b^
Khan [30]	↑^e,f^	NR	↑ liver histology score^a,b^	ND 6 weeks of diet (lung; U/mg tissue) LFD: 51.2 ± 3.38 HFD-S: 46.9 ± 2.20 ↓ 15 weeks of diet (lung; U/mg tissue)^a,d^ LFD: 44.1 ± 2.86 LFD-DR: 63.2 ± 5.60 HFD-S: 26.3 ± 3.80 ↓ 27 weeks of diet (lung; U/mg tissue)^a,d^ LFD: 47.5 ± 2.70 LFD-DR: 43.9 ± 3.29 HFD-S: 28.3 ± 5.08	↑ 6 h (liver)^b^ ND 6 h (liver)^a^	ND 6 h (liver)
Lewis [31]	↑^e^ 6h^b^, 24h^b^	↓ LFD-S: 75% HFD-S: 33%	↑creatinine 24 h^a,^^b^ (plasma) ↑ NGAL 24 h^a,^^b^ (kidney) ↑ ketones 6 h^b^, 12 h^b^, 36 h^b^, 48h^b^ (blood)	NR	↓ 6 h (plasma)^a^ ND 24 h (plasma)^a^ ↑ 24 h (plasma)^b^	ND 24 h^a^ (plasma) ↑ 24 h^b^ (plasma)
Panpetch [32]	↑^e^	ND	↑ ALT (serum) 24 h^b,c^ ↑ creatinine 24 h^a^^,b,c^ (serum)	NR	↑ (serum) 24 h^a,^^b,c^	↑ (serum)^a,b,c^
Rivera [33]	NR	NR	NR	NR	NR	↑ (liver) 6 h^a,^^b^
Siegl [34]	NR	↑	↓interstitial and alveolar edema 24 h^a^, 48 h^a^ ND: liver histology	NR	↑ (serum; ng/ml) 6 h^b^: LFD-S: 20.8 ± 2.2 HFD-S: 18.2 ± 2.6	↓ (serum; ng/ml)^a^ 24 h: LFD-S: 4.2 ± 1.0 HFD-S: 1.3 ± 0.1 48 h: LFD-S: 5.7 ± 0.9 HFD-S: 3.0 ± 0.7
Souza [35]	NR	↑	NR	NR	↑ hypothalamus^a,b^	↑ hypothalamus^a^
Strandberg [36]	NR	↑	NR	NR	ND 5–7 days (serum, liver, spleen)	↑ ND 5–7 days (liver, spleen, serum)
Su [37]	NR	NR	↑BUN (mg/dL; plasma)^c^: LFD:18.9 ± 0.90 12 h: HFD-S: 67.4 ± 8.40 24 h: HFD-S: 97.1 ± 6.10 48: HFD-S: 139.9 ± 14.40 ↑ Creatinine (mg/dL; plasma)^c^: LFD: 0.09 ± 0.01 12 h: HFD-S: 0.14 ± 0.03 24 h: HFD-S: 0.70 ± 0.09 48 h: HFD-S: 1.10 ± 0.28 ↑ NGAL (ug/dL; plasma)^c^: LFD: 0.08 ± 0.01 12 h: HFD-S: 3.52 ± 1.84 24 h: HFD-S: 52.3 ± 5.40 48 h: HFD-S: 39.5 ± 32.70	↑ 12 h^c^, 14 h^c^, 48 h^c^ (kidney)	↑12 h^c^, 24^c^, 48 h^c^ ( kidney)	↑12 h^c^, 24^c^, 48 h^c^ (kidney)
Vankrunkelsven [38]	↑ glucose^e^ ~ 85 h^c^, ~ 125 h^c^	ND	↑ketones (blood)^c^ ↓ liver edema 125 h^c^ ↓ BUN (plasma)^c^	NR	↑ (plasma)^b^	↑ (plasma)^b^
Williamson [39]	NR	NR	ND ALT 18 h (plasma; U/L) LFD-S:118 ± 33 HFD-S:102 ± 45	↑ 18 h (lung)^a,b^ HFD: 53 ± 22 HFD-S: 124 ± 31 LFD-S: 84 ± 29 ND 18 h (liver)	NR	NR
Yeh [40]	NR	NR	NR	↑ 12 h, 24 h (lung)^b^	NR	NR

*HFD-S* high-fat diet septic, *LFD-S* low-fat diet septic, *LFD-DR* low-fat diet, diet restricted, *ND* no difference between HFD-S group and any other group, *AU* arbitrary units, *ALT* alanine transaminase, *BUN* blood urea nitrogen, *NGAL* neutrophil gelatinase-associated lipocalin, *cTnI* cardiac troponin, *NR* not reported

↑ = increase

↓ = decrease

^a^HFD-S vs LFD-S

^b^HFD-S vs HFD

^c^HFD-S vs LFD

^d^HFD-S vs LFD-DR

^e^HFD vs LFD

^f^HFD vs LFD-DR

^g^U/mg tissue

^h^Measured in serum

^i^Prior to sepsis induction

Liver impacts were explored in three (18%) studies through histology [22, 26, 30]. Two (67%) indicated greater liver damage in obese septic mice compared to non-obese septic mice [22, 26], but one (33%) found no distinctions [30]. Alanine transaminase (ALT) findings were inconsistent among four (24%) studies [22, 25, 28, 35]. Two (50%) reported no differences between obese and non-obese septic mice, whether in serum [22] or plasma at 18 h [35]. Conversely, one (25%) study showed elevated plasma ALT in obese septic mice compared to non-obese mice post-sepsis and obese non-septic mice at 6 h [25], and another showed increased serum ALT in obese septic mice compared to obese and non-obese mice 24-h post-sepsis [28]. Myeloperoxidase (MPO), a damage surrogate [37], was assessed in three (18%) studies [24, 25, 35]. Two (67%) saw higher liver MPO levels in obese septic mice at 6-h post-sepsis compared to non-obese septic mice [24, 25], and one showed an increase at 18 h [24]. One (33%) found no MPO differences at 18 h [35]. Among three (18%) studies measuring liver IL-6 levels [22, 26, 32], no distinctions were seen between obese and non-obese septic mice at 6 h [26]. Yet, two (67%) showed significant differences between obese septic and non-septic mice, at 6 h [22] and 7 days [26], while one (33%) found no differences among any cohort at 5–7 days [32]. Similarly, two (12%) studies detected no differences in hepatic TNFɑ between septic groups at 6 h [26] or 4–7 days [32]. However, two (12%) studies noted higher levels in obese septic mice compared to obese non-septic mice at 6 h [29] and 7 days [22].

Histological evaluation of lungs occurred in two (12%) studies [24, 30]. One study found no inflammation at 6-h post-sepsis in both obese and non-obese mice, noting interstitial and alveolar edema increase at 24 and 48 h in non-obese septic mice compared to obese septic mice [30]. Conversely, the other study showed higher lung injury scores in obese septic mice at 6 h, with alveolar congestion, hemorrhage, neutrophil infiltration, and aggregation, and hyaline membrane formation [24]. Lung MPO levels were assessed in four studies (24%) [24, 26, 35, 36]. One study showed increased MPO in obese mice at 1-, 6-, and 18-h post-sepsis compared to non-obese mice [24]. Another noted MPO elevation at 12 and 24 h in obese septic mice compared to obese non-septic mice [36], and a third observed MPO increase at 18 h in obese mice compared to non-obese mice post-sepsis and obese non-septic mice [35]. The fourth study found higher MPO in non-obese septic mice than obese septic mice at 6-h post-sepsis after 15- or 27-week diets, with no difference after 6 weeks [26]. No studies reported lung IL-6 or TNFɑ levels.

Biomarkers for kidney damage, including creatinine, neutrophil gelatinase-associated lipocalin (NGAL), and blood urea nitrogen (BUN) were assessed. Creatinine levels were evaluated in three (18%) studies [27, 28, 33]; two saw an increase in obese septic mice compared to non-obese septic controls at 24-h post-sepsis in plasma [27] and serum [28], while the third [33] found an increase in plasma creatinine levels in obese septic mice at 12-, 24-, and 48-h post-sepsis compared to non-obese non-septic mice. NGAL, evaluated in two (12%) studies, increased in the kidney tissue of obese septic mice compared to non-obese septic mice at 24 h [27] and in the plasma of obese septic mice compared to non-obese non-septic mice at 12, 24 and 48 h [33]. Plasma BUN levels were measured in two studies (12%); one found it increased at 12-, 24-, and 48-h post-sepsis in obese septic mice compared to non-obese non-septic mice [33] but decreased in another [34]. One (6%) study also showed increased IL-6 levels in obese septic mice compared to non-obese non-septic mice at 12-, 14- and 48-h post-sepsis [33]. TNFɑ levels in the kidney were not reported in any study.

### Risk of bias results

**Fig. 2:**
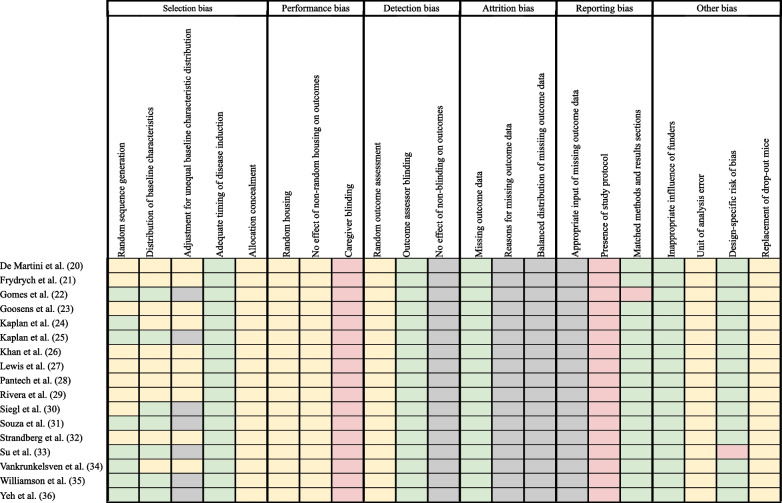
Risk of bias results for each individual study evaluated using a modified SYRCLE tool. Red squares indicate high risk, green squares indicate low risk, and yellow squares represent unclear risk, and grey squares indicate not applicable

The risk of bias in the studies was assessed using a modified version of the SYRCLE tool, consisting of 21 sub-items as signaling questions (Fig. 2). Responses of "yes" indicated low risk, "no" indicated high risk and "unclear" indicated unclear risk. Across all studies, two sub-items were deemed high risk (9.5%), six were unclear risk (29%), while four (19%) were categorized as low risk. High-risk sub-items included "caregiver blinding" due to visual differentiation between obese and non-obese mice and "presence of study protocol," as no study had a registered protocol. For the "random sequence generation" sub-item, eight studies were marked as low risk as they mentioned animal randomization but lacked a method description. In almost all cases of unclear risk sub-items, it was impossible to evaluate due to insufficient reporting in the methods sections. However, for the “distribution of baseline characteristics” sub-item, two studies [24, 28] were evaluated as unclear risk as baseline weight data was shown graphically but not described explicitly. Sub-items that were considered low risk in all studies pertained to “adequate timing of disease induction,” as outcome assessment methods were the same for both obese and non-obese mice; “missing outcome data,” as this was not assumed unless explicitly stated; “outcome assessor blinding,” as all animals were evaluated for all outcomes; and “inappropriate influence of funders,” as determined by examining funding and disclosure statements. The “matched methods and results” sub-item was low risk in all studies except Gomes et al. [22] which did not report results associated with chemokine ligand 2, despite being mentioned in the methods. The “design-specific risk of bias” sub-item was low risk in all studies except Su et al. [33], as it did not induce sepsis in non-obese mice.

## Discussion

Sepsis, a life-threatening condition, is influenced by obesity, but its impact remains inconclusive, possibly showing a survival benefit within a specific weight range [38]. A prior review [39] assessed obesity's effect on murine sepsis survival and organ injury using diverse animal models, complicating the synthesis and interpretation of its translational relevance. This scoping review aimed to clarify outcomes in murine models involving DIO and pathogen-induced sepsis. The primary aim was to identify the reported variables in current sepsis and obesity literature. Within included studies, disparities in observed outcomes, divergent evaluated outcomes, methodological variations, and limitations in sepsis and obesity models were identified. Few studies reported mortality, lacking consensus on whether murine models support or contradict the clinically observed obesity paradox. Inconsistent results extended to parameters, such as histological lung and liver damage evaluations, with reported outcomes varying from organ dysfunction to inflammatory cytokines. Diverse outcome investigation compounded result synthesis difficulties. Methodological disparities, including sepsis induction methods and specific high-fat and control diets, hindered comparisons even among studies evaluating similar outcomes. The sepsis and obesity models suffered limitations: sepsis standard misalignment, improper control diets, unstandardized murine obesity criteria, and lack of consideration of experiment timing and season. These limitations contributed to result variability. Furthermore, the lack of inclusion of both sexes limits generalizability. For these reasons, the secondary objective to determine whether DIO offers protection against sepsis-induced organ dysfunction could not be achieved, due to a lack of consensus on the effects of obesity and sepsis. Given the significant variability in various aspects of pre-clinical models related to sepsis and obesity, summarized in Table 5, this scoping review pinpointed crucial elements that need consensus within the broader field to improve outcome comprehension.

**Table 5:** Overview of variables, consensus points, and possible solutions

	Variable	Consensus points or potential solutions
**Obesity Models**	Biological sex	Incorporate both male and female mice
	Characterization of obesity	Use one or more of the following: • Body weight • Body composition • Glucose tolerance test • Insulin resistance test
	Diet	Use ingredient matched to control diets Ideal source of fat or fat percentage of diet Source and percentage of carbohydrates Diet restriction vs ad libitum Length on diet
	Seasonality	Include study seasons in the methodology
	Rodent husbandry	Include housing details (e.g., cage type, bedding, room temperature) in the methodology
**Sepsis Models**	Biological sex	Incorporate both male and female mice
	Model	• Cecal ligation and puncture • Fecal induced peritonitis • Bacterial isolates • Respiratory tract infection • Urinary tract infection
	Dose of inoculum	Ideal dosage for outcomes, i.e. single versus double puncture model with a specific needle gauge
	Fluid resuscitation	Type: • Saline • Lactated ringers Dose Timing of administration
	Antibiotic administration	Type • Imipenem/Cilastan • Piperacillan-tazobactum Dose Timing of administration • Early versus late antibiotic administration
	Analgesic administration	Type • Buprenorphine • Fentanyl Dose Timing of administration
	Seasonality	Include study seasons in the methodology
**Reported Outcomes**	Mortality	Report mortality or explicitly mention its absence in applicable models
	Lung dysfunction	• Blood gases • Myeloperoxidase
	Liver dysfunction	• Alanine transaminase (ALT) • Aspartate aminotransferase (AST) • Bilirubin • Myeloperoxidase
	Kidney dysfunction	• Creatinine • Neutrophil gelatinase-associated lipocalin (NGAL) • Blood urea nitrogen (BUN) • Cystatin C
	Histology	Include histology for structural and morphological information but avoid relying solely on it for assessing dysfunction

A 2017 global study found higher age-standardized sepsis incidence in females than males [1], but all studies reviewed used only male mice except one, limiting translational value. Differences in myocardial and immune responses between male and female mice emphasize the need for both sexes in sepsis research [40]. Biological sex impacts obesity, with distinct adipose patterns and metabolic traits; in particular, women generally have more subcutaneous adipose tissue (SAT); while men have greater visceral adipose tissue (VAT) [41]. Increased VAT in men has been associated with worse glucose, lipid, and inflammatory outcomes than women [42, 43]. In addition, a high VAT/SAT ratio has been shown to influence sepsis survival negatively [44]. Additional mechanisms that are known to be impacted by both obesity and sepsis in a sex-dependent manner including the impact of nitric oxide on vasomotor tone and function should also be considered. Estrogen has been shown to enhance nitric oxide production, which is impaired by both obesity [45] and sepsis [46]. The nitric oxide pathway is a crucial factor that, to date, has been examined independently. However, the existing literature strongly supports further investigation within a co-morbidity model encompassing both sepsis and obesity. Investigating sex's role in the interplay between sepsis and obesity is crucial due to their sex-dependent variations.

The translational applicability of the studied murine sepsis models was diminished due to a lack of alignment with current clinical standards. The Surviving Sepsis Campaign, a set of international guidelines for sepsis clinical care, recommends antibiotic administration within 1 h for patients with septic shock or suspected sepsis with shock and within 3 h for suspected septic patients without shock [47]. The Minimum Quality Threshold in Pre-Clinical Sepsis Studies (MQTiPSS), recommendations developed by an expert group to improve animal models of sepsis, considers fluid administration essential [48, 49]. In contrast, many evaluated studies did not provide antibiotics or fluid resuscitation throughout the sepsis course. Accounting for the six studies published after MQTiPSS fluid administration guidelines were published in 2019, only four studies reported fluid administration [22, 33, 34, 36], while only one reported the use of antibiotics [34]. Clinical sepsis treatment, based on physiological parameters, differs from immediate administration in murine models [27]. Among the reviewed studies, the timing of antibiotic administration differed, possibly due to a lack of characterization of the difference in temporal kinetics between clinical and murine sepsis, as the condition progresses much faster in mice than in humans [50]. Antibiotic timing variations can impact outcomes, influenced by differences in sepsis progression between mice and humans. Administering antibiotics too early in murine models may hinder proper illness induction, affecting host response. Delayed antibiotic administration post-sepsis induction has shown different mortality rates and pathology outcomes. It has been shown in a cecal slurry model that providing antibiotics at 1- or 6-h post-sepsis induction showed low mortality and did not lead to sepsis-associated pathology while delaying antibiotic administration to either 12- or 16-h post-sepsis induction led to higher mortality [51].

The variability in diets used across studies presents challenges in determining the exclusive impact of a high-fat diet versus ingredient-related effects. Control diets are often vaguely labeled as "normal" or "standard" chow, with differing compositions of refined and unrefined plant ingredients [52]. This leads to variations in dietary fiber, with refined diets lacking soluble fiber that promotes beneficial bacterial growth, potentially leading to disruptions in colonic microbiota and obesogenic effects [53]. One option in DIO studies is to use control diets matched in the types of nutritional ingredients to the high-fat diet [54]. A high-fat and low-fat diet, matched in composition, both showed an increased Fimircutes:Bacteriodetes ratio and reduced diversity in the intestinal microbiota compared to the chow diet, but still maintained differences in body weight and fat mass between diet cohorts [52]. However, caution is needed if a matched control diet uses sugar as a fat-derived calorie source, as this could impact observations. The intricate interplay between the gut microbiota and immune responses adds complexity to studying conditions, such as sepsis and obesity [55], emphasizing the need for careful diet selection.

All but three studies used CLP for sepsis induction. Although this is the current gold standard in murine sepsis studies, this method has issues, such as high inter-operator variability and challenges in standardizing between individual mice [56]. In DIO research, CLP's reliance on cecal contents exacerbates variability. CLP often lacks characterization of cecal matter composition, potentially overlooking confounding effects [57]. Fecal-induced peritonitis (FIP) a newer model, offers better reproducibility without CLP's technical challenges, but lacks a continuous polymicrobial focus as seen in appendicitis/diverticulitis, leading to an intense initial immune response that does not reflect sepsis-associated hemodynamic and metabolic changes [58]. Nonetheless, FIP worsens outcomes dose-dependently, upregulating pro-inflammatory gene expression such as chemokine ligand 2 and interleukin-6 [56]. FIP and CLP display similar physiological, histopathological, and immunological alterations similar to observed clinical sepsis alterations with FIP showing less variation [59]. This review highlights the prevalent focus on abdominal sepsis in pre-clinical models. It is crucial to broaden investigations to include other clinically relevant sepsis models, especially those in the obese population from respiratory and urinary origins [60]. The selection of models that mimic clinical features while ensuring benchmarks for reproducibility is essential for inter-laboratory comparisons.

The characterization of obesity in numerous studies varied significantly, assessed through body weight, body composition, glucose tolerance, and insulin tolerance. Weight measurement alone overlooks body composition differences. For example, a low-carbohydrate, high-fat diet, compared to standard chow, elicited similar weight gain, but showed a decrease in lean mass and organ deterioration [61]. In addition, as observed in our review, glucose tolerance tests (GTTs) differed in glucose administration route and fasting duration, convoluting comparisons. Intraperitoneal (IPGTT) and oral gavage (OGTT) tests show differing insulin levels and glucose release patterns [62]. Obesity in humans is categorized primarily according to body mass index (BMI); however, there are no corresponding criteria for mice [12]. Proposed murine obesity characterization combines weight, composition, inflammation, glucose, liver health, hormones, and lipids [63]. The absence of standardized obesity criteria hinders accurate sepsis–obesity effect investigations.

Seasonal and daily times of sepsis induction can also determine sepsis outcomes, but the time of day in which sepsis was induced was only reported in one study [30]. Among clinical cases of sepsis, winter has been associated with higher incidence and mortality than summer [64]. Even in consistently maintained conditions of animal facilities, mice subjected to CLP have been shown to exhibit season-dependent outcomes [65, 66]. Both male and female C57BL/6 J mice that underwent CLP show circadian rhythm-dependent severity—mortality is higher when sepsis is induced at night compared to the day [67, 68]. Due to this, future studies should evaluate seasonality as an experimental factor in murine models of sepsis and obesity.

This study is subject to several important limitations. Firstly, the exclusion of studies without measures of organ dysfunction restricts the inclusion of mechanistic investigations. Secondly, the generalizability of our findings is limited by focusing solely on murine models. Omitting diverse preclinical models, such as rats and pigs, may constrain translational relevance and study generalizability. Thirdly, the inclusion criterion of English-language studies may have excluded relevant non-English publications. Despite these limitations, the review adheres to standardized PRISMA–ScR guidelines [16], and its inclusion of risk of bias assessments highlights methodological considerations essential for addressing translational challenges in animal models of sepsis. The review effectively underscores methodological inconsistencies and knowledge gaps in murine sepsis–obesity models that require resolution for advancing research. Moreover, the review's identification of reported outcomes in these models offers valuable insights for developing a standardized set of reportable outcomes for future studies advancing comparability to synthesize outcomes.

## Conclusion

The absence of co-morbidity representation, particularly obesity, in pre-clinical sepsis studies has impeded their translation into effective treatments, resulting in conflicting data and methodological inconsistencies that hinder consensus and applicability. To address the complexity of sepsis, utilizing various animal models that replicate clinically observed sepsis is crucial. Despite inherent limitations, this review underscores the importance of standardized protocols to synthesize the impact of obesity on sepsis outcomes. Collaborative initiatives such as the National Pre-clinical Sepsis Platform are striving to establish uniform practices and comparability across laboratories [69, 70]. Standardization in murine sepsis–obesity models will enhance insights into pathophysiology and improve pre-clinical therapeutic translation.

### Supplementary Information

**Additional file 1: **Example search strategy.

**Additional file 2: Table S1. **Modified SYRCLE risk of bias signaling questions.

## Data Availability

Source data for this study are available through the corresponding author.
