# Correction: Sepsis and obesity: a scoping review of diet-induced obesity murine models

**DOI:** 10.1186/s40635-024-00637-4

**Published:** 2024-06-05

**Authors:** Mikaela Eng, Keshikaa Suthaaharan, Logan Newton, Fatima Sheikh, Alison Fox-Robichaud, the National Preclinical Sepsis Platform, Sepsis Canada

**Affiliations:** 1 Thrombosis and Atherosclerosis Research Institute (TaARI), Hamilton, Canada; 2 Department of Medicine, Faculty of Health Sciences, McMaster University, Hamilton, Canada; 3 Department of Health Research Methods, Evidence and Impact, Faculty of Health Sciences, McMaster University, Hamilton, Canada; 4 Division of Critical Care, Department of Medicine, Faculty of Health Sciences, McMaster University, Hamilton, Canada

**Correction: Intensive Care Medicine Experimental (2024) 12:15** 10.1186/s40635-024-00603-0

Following publication of the original article [1], it came to the attention of the journal that Table 5 had been omitted from the article. The table has since been added to the article. The publisher thanks you for reading and apologizes for any inconvenience caused.
