# Supplementary material for: Sepsis and obesity: a scoping review of diet-induced obesity murine models
Source: Intensive Care Med Exp. 2024 Feb 23;12:15. doi: 10.1186/s40635-024-00603-0 (PMC10884395; doi:10.1186/s40635-024-00603-0)
Supplement: Supplementary file 1 — Additional file 1: Example search strategy. [file 40635_2024_603_MOESM1_ESM.docx]

**Additional file 1:** Example search strategy

Ovid MEDLINE(R) ALL <1946 to June 15, 2023>

1 Sepsis/ (67754)

2 septicemia.mp. or Sepsis/(76839)

3 Bacteremia/(27177)

4 septic shock.mp. or Shock, Septic/(38490)

5 Diet/ or Diet, High-Fat/ or Obesity/(384102)

6 murine.mp/(258924)

7 mouse.mp. or Mice/(1825589)

8 1 or 2 or 3 or 4 (131077)

9 6 or 7 (1880037)

10 5 and 8 and 9 (57)
