# Supplementary material for: Sepsis and obesity: a scoping review of diet-induced obesity murine models
Source: Intensive Care Med Exp. 2024 Feb 23;12:15. doi: 10.1186/s40635-024-00603-0 (PMC10884395; doi:10.1186/s40635-024-00603-0)
Supplement: Supplementary file 2 — Additional file 2: Table S1. Modified SYRCLE risk of bias signaling questions. [file 40635_2024_603_MOESM2_ESM.docx]

| Type of bias | Signaling question |
| --- | --- |
| Sequence Generation (Selection bias) | Was the allocation sequence adequately generated and applied? (Yes/No/Unclear) |
| Baseline characteristics (Selection bias) | Was the distribution of relevant baseline characteristics balanced for the intervention and control groups? (Yes/No/Unclear) |
|  | If relevant, did the investigators adequately adjust for unequal distribution of some relevant baseline characteristics in the analysis? (Yes/No/Unclear) |
|  | Was the timing of disease induction adequate? (Yes/No/Unclear) |
| Allocation concealment (selection bias) | Could the investigator allocating the animals to intervention or control group not foresee assignment due to one of the following or equivalent methods? (Yes/No/Unclear) |
| Random housing (Performance bias) | Did the authors randomly place the cages or animals within the animal room/facility? (Yes/No/Unclear) |
|  | Is it unlikely that the outcome or the outcome measurement was influenced by not randomly housing the animals? (Yes/No/Unclear) |
| Blinding (performance bias) | Was blinding of caregivers and investigators ensured, and was it unlikely that their blinding could have been broken? (Yes/No/Unclear) |
| Random assessment (detection bias) | Did the investigators randomly pick an animal during outcome assessment, or did they use a random component in the sequence generation for outcome assessment? (Yes/No/Unclear) |
| Blinding (detection bias) | Was blinding of the outcome assessor ensured, and was it unlikely that blinding could have been broken? (Yes/No/Unclear) |
|  | Was the outcome assessor not blinded, but do review authors judge that the outcome is not likely to be influenced by lack of blinding? (Yes/No/Unclear) |
| Selective outcome reporting (reporting bias) | Were all animals included in the analysis? (Yes/No/Unclear) Assume yes unless otherwise stated |
|  | Were the reasons for missing outcome data unlikely to be related to true outcome? (e.g., technical failure) (Yes/No/Unclear) |
|  | Are missing outcome data balanced in numbers across intervention groups, with similar reasons for missing data across groups? (Yes/No/Unclear) |
|  | Are missing outcome data imputed using appropriate methods? (Yes/No/Unclear) |
| Selective outcome reporting | Was the study protocol available and were all of the study’s pre-specified primary and secondary outcomes reported in the current manuscript? (Yes/No/Unclear) |
|  | Was the study protocol not available, but was it clear that the published report included all expected outcomes (i.e. comparing methods and results section)? (Yes/No/Unclear) |
| Other biases | Was the study free of inappropriate influence of funders? (Yes/No/Unclear) |
|  | Was the study free of unit of analysis errors? (Yes/No/Unclear) |
|  | Were design-specific risks of bias absent? (Yes/No/Unclear) |
|  | Were new animals added to the control and experimental groups to replace drop-outs from the original population? (Yes/No/Unclear) |

**Table S1:** Modified SYRCLE risk of bias signaling questions
